# Influence of toileting behavior on the natural course of anterior vaginal wall prolapse

**DOI:** 10.1186/s12905-022-01637-w

**Published:** 2022-03-03

**Authors:** Osman Kose, Yavuz Tarik Atik, Deniz Gul, Burak Uysal, Haci Ibrahim Cimen, Mehmet Suhha Bostanci

**Affiliations:** 1 Department of Urology, Training and Research Hospital, Faculty of Medicine, Sakarya University, Sakarya, Turkey; 2 Department of Gynecology and Obstetrics, Faculty of Medicine, Sakarya University, Sakarya, Turkey

**Keywords:** Pelvic organ prolapse, Squatting, Toileting behavior

## Abstract

**Background:**

Many risk factors for pelvic organ prolapse (POP) have been proposed, and the cause is most likely multifactorial. This study aimed to investigate the effect of toileting behaviors on the natural course of anterior vaginal wall prolapse (AVWP).

**Methods:**

Data on 75 women who underwent surgery for symptomatic AVWP were collected. Patients with grade ≥ II AVWP were included in this study and were divided into two groups according to their voiding and defecation position. The volunteers who voided and defecated in a sitting position comprised Group 1, and those who voided and defecated in a squatting position comprised Group 2. The Colorectal-Anal Impact Questionnaire (CRAIQ), Pelvic Floor Impact Questionnaire (PFIQ), Pelvic Organ Prolapse Impact Questionnaire (POPIQ), Urinary Impact Questionnaire (UIQ) and visual analog scale (VAS) pain scores were used to evaluate the patients’ symptoms.

**Results:**

Forty-four patients were included in Group 1 (sitting position), and 31 patients were included in Group 2 (squatting position). The groups were similar in terms of BMI, parity, menopause duration, topical estrogen use, comorbidities, the presence of constipation and urinary incontinence, and the pad count for incontinence. The time from initial symptoms to surgery was shorter in Group 2 than in Group 1 12 (3–73) and 24 (2–182) months (p = 0.001), respectively. The PFIQ, POPIQ and POP-related VAS scores were significantly higher in patients who voided and defecated in a squatting position.

**Conclusion:**

In patients with symptomatic POP, increased IAP while performing the squat position during defecation and voiding may increase the severity of patients' symptoms related to prolapse more than that of sitting position. Therefore, questioning the toileting position of patients with AVWP may help inform management decisions, with changing to a sitting position encouraged.

## Background

Pelvic organ prolapse (POP) is defined as the downward descent of female pelvic organs (bladder, uterus or posthysterectomy vaginal cuff, and the small or large bowel) into or through the vagina; it affects millions of women worldwide and is increasingly recognized as a global burden to women’s health [1]. The social, psychological, and economic costs of POP can be high. Approximately 11.8% of women will need surgery for POP, urinary incontinence, or both during their lifetime [2]. A significant number of these patients will undergo two or more challenging surgical procedures. Most often, the anterior vaginal wall is the prolapsing part of the vaginal canal, and it is also the part most likely to fail in the long term after surgical correction [2].

Many etiological risk factors for the development of POP have been postulated. Performing activities that lead to increased intra-abdominal pressure (IAP) is often cited as a potential cause of POP [3]. Although little is known about the natural history of POP, clinical practices are guided by the assumption that POP is a progressive disease [2]. The Squat is the exercise that mostly increases IAP among high-intensity interval exercises [4]. Even air squatting performed without weights is considered a strenuous cross-fit movement that leads to increased IAP [4].

Squatting remains the traditional position for voiding and defecating in Asia (including Japan, Korea, and China) and Africa. The Western population, on the other hand, has gotten used to sitting on toilet seats. In Westernized countries, squatting and sitting positions are also used for defecation and voiding. Researchers have become more interested in women’s toilet behaviors in recent years. There are many studies on the effect of toilet position on voiding and defecation physiology [5, 6]. In contrast, information on the impact of toilet position on the natural course of POP is limited. This study aimed to investigate the effect of toileting behaviors on the natural course of anterior vaginal wall prolapse (AVWP).

## Material and methods

**Fig. 1:**
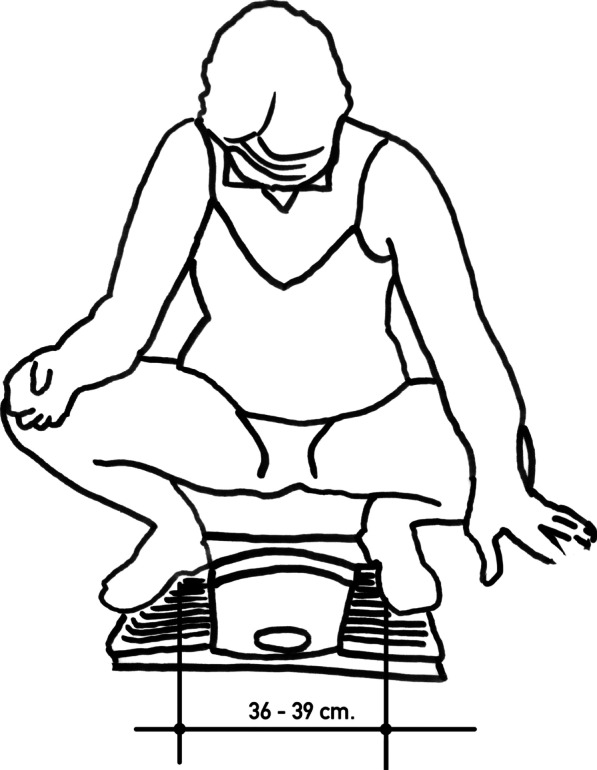
Squatting position

**Fig. 2:**
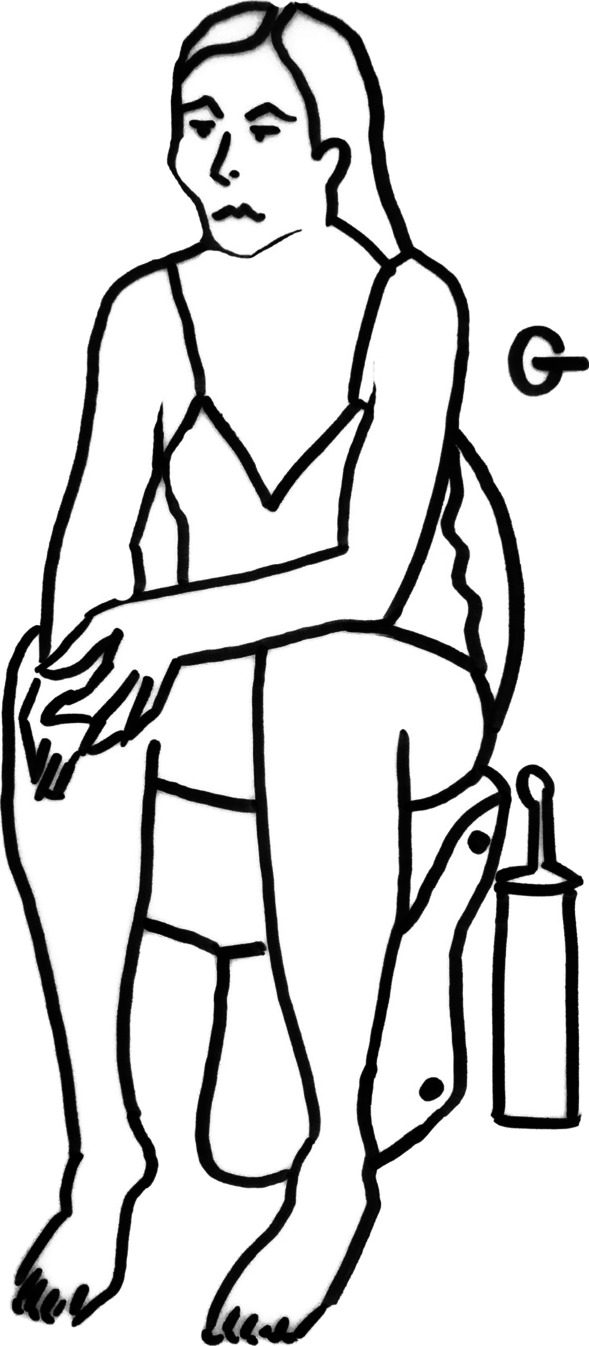
Sitting position

We performed a retrospective review of a prospectively acquired database of 75 women who underwent surgery for symptomatic AVWP, from March 2015 to February 2021 (the Sakarya University Faculty of Medicine institutional ethics committee number of E-71522473-050.01.04-14814-94, date of 15/02/2021). Patients with grade ≥ II AVWP were included, and they were divided into two groups according to voiding and defecation position. The volunteers who voided and defecated in a sitting position comprised Group 1, and the volunteers who voided and defecated in a squatting position comprised Group 2. The squatting and sitting positions are depicted in Figs. 1 and 2, respectively. After obtaining ethics committee approval, the toileting positions of the patients included in the study were determined by asking the patients via telephone call or in the outpatient clinic. The Pelvic Organ Prolapse Quantification System (POP-Q), described previously in the literature, was used, and the results were recorded for each patient in the dorsal lithotomy position at the time of the decision to operate [7]. The Pelvic Floor Impact Questionnaire-7 (PFIQ-7) was used to evaluate the patients’ symptoms, along with data collected by interview at the outpatient clinic at the time of the decision to operate. The PFIQ-7 has been validated in Turkish and is a reliable and easy-to-use questionnaire on the condition-specific quality-of-life of women with pelvic floor disorders. The PFIQ-7 presents seven questions with three subscales: the Urinary Impact Questionnaire (UIQ-7), Colorectal–Anal Impact Questionnaire (CRAIQ-7), and Pelvic Organ Prolapse Impact Questionnaire (POPIQ-7), with four answers for each column (not at all = 0; somewhat = 1; moderate = 2; a lot = 3). The final score was calculated by multiplying the total of the subscale scores by 33.3, and the total value ranged from 0 to 300 [8].

Demographic data, visual analog scale (VAS) pain scores, and questionnaire scores were recorded. Patients with prior urogynecology operation history, neurologic disorders, and immobility were excluded from the study. In addition, patients with a diagnosis of urge urinary incontinence and overactive bladder before the onset of POP symptoms were also excluded from the study. All methods were performed in accordance with the relevant guidelines and regulations. Informed consent was obtained from all subjects.

### Statistical analysis

Statistical analyses were performed using SPSS 21.0 (IBM, NY, USA). The Kolmogorov–Smirnov test was used to evaluate whether the data followed a normal distribution. The mean and standard deviation (mean ± SD) are used to report continuous variables with normal distributions, whereas the median (min–max) is used to report variables with nonnormal distributions. Categorical variables are shown as the number of cases (n) and percentage (%). Independent samples t tests or Mann–Whitney U tests were used to compare continuous data, and chi-square tests or Fisher's exact tests were used to compare categorical data. p < 0.05 was considered statistically significant.

## Results

**Fig. 3:**
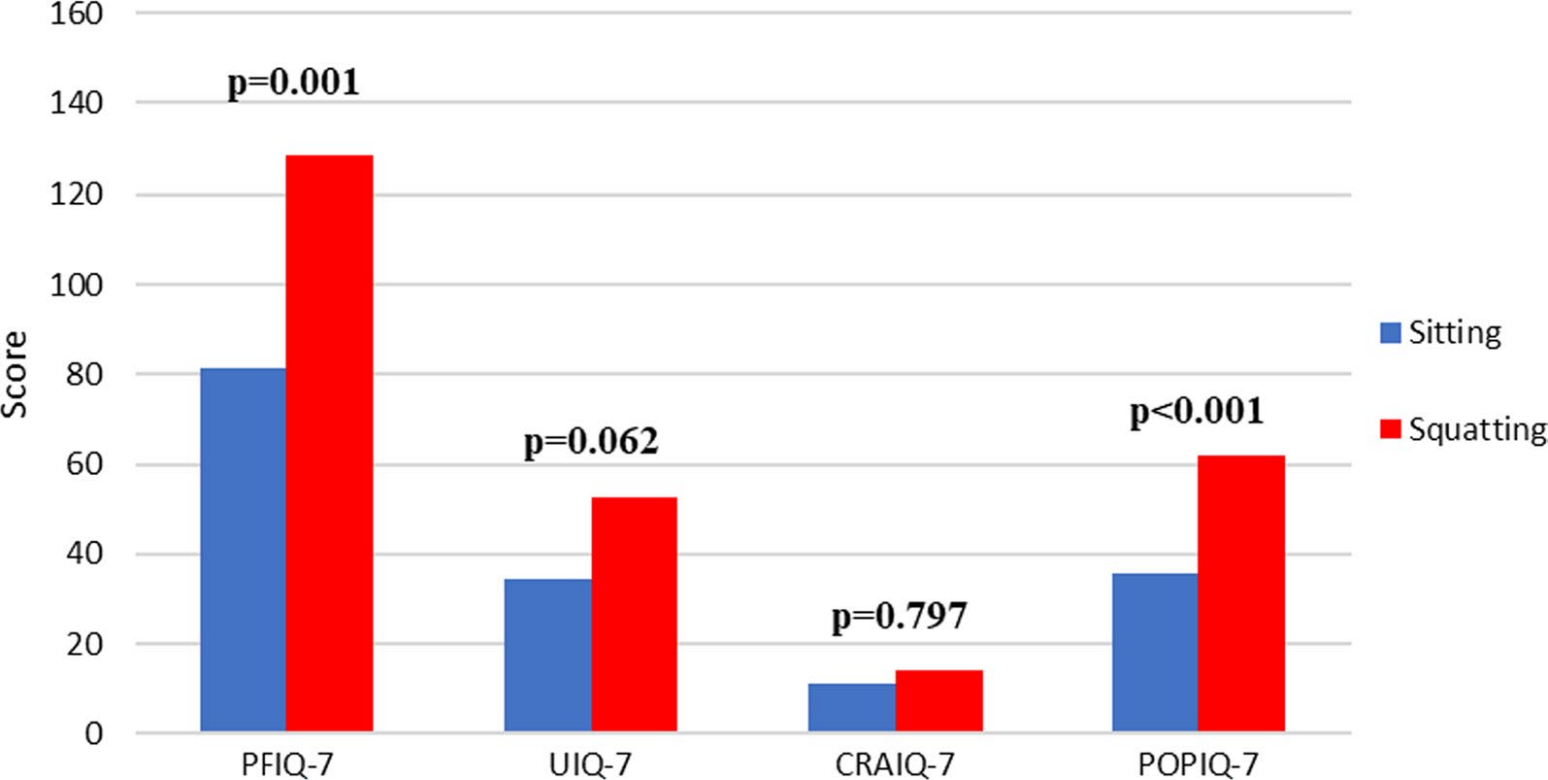
Comparison of the Pelvic Floor Impact Questionnaire total and subscale scores between groups

In total, 44 patients were enrolled in Group 1 (sitting position), and 31 patients were enrolled in Group 2 (squatting position). The mean age was 57.84 ± 9.19 in Group 1 and 58.29 ± 9.07 in Group 2 (p = 0.835). The groups were similar in terms of body mass index (BMI), parity, duration of menopause, topical estrogen use, comorbidities, the presence of constipation and urinary incontinence, and the pad count for incontinence; this is shown in Table 1. All of the patients in both groups were housewives who did not perform strenuous work such as lifting sofa or bed. The POP-related VAS score was significantly higher in Group 2 than in Group 1: 2 (0–7) and 5 (0–10) (p < 0.001) for Group 1 and Group 2, respectively. The time from initial symptoms to surgery was longer in Group 1 than in Group 2: 24 (2–182) and 12 (3–73), respectively (p = 0.001) (Table 1). There was no difference in POP-Q measurements between the groups (Table 2). The C point was − 4 (− 1, − 7) in Group 1 and − 5 (− 2, − 6) in Group 2 (p = 0.847) (Table 2). While the PFIQ and POPIQ scores were significantly higher in patients who voided and defecated in squatting positions (Group 1), the CRAIQ and UIQ scores were similar between groups (Fig. 3).

**Table 1:** Comparison of demographic and preoperative data according to voiding and defecation position

	Group-1 (sitting) (n = 44)	Group-2 (squatting) (n = 31)	p
Age (year) (mean ± SD)	57.84 ± 9.19	58.29 ± 9.07	0.835^a^
BMI (kg/m^2^) (mean ± SD)	29.17 ± 3.28	29.67 ± 2.98	0.505^a^
Parity (n) (median) (min–max)	3 (1–5)	3 (2–8)	0.221^b^
VAS score (median) (min–max)	2 (0–7)	5 (0–10)	< 0.001^b*^
Pad count (n) (median) (min–max)	2 (0–6)	2 (0–6)	0.731^b^
Menopause duration (month) (median) (min–max)	125 (1–360)	120 (5–300)	0.475^b^
Time to surgery (month) (median) (min–max)	24 (2–182)	12 (3–73)	0.001^b*^
Topical estrogen use, yes (n) (%)	4 (9.1)	6 (19.4)	0.173^c^
Comorbidities (n) (%)			
DM	11 (25)	6 (19.24)	0.565^d^
HT	22 (50)	17 (54.80)	0.680^d^
CAD	2 (4.5)	1 (3.2)	0.630^c^
CHF	0 (0)	2 (6.5)	0.168^c^
COPD	1 (2.3)	2 (6.5)	0.370^c^
Menopause, yes (n) (%)	34 (77.3)	27 (87.1)	0.282^d^
Smoking, yes (n) (%)	5 (11.4)	7 (22.6)	0.162^c^
Symptoms, yes (n) (%)			
Vaginal bulging	37 (84.1)	30 (96.8)	0.081^c^
Urgency	19 (43.2)	18 (58.1)	0.204^d^
Incontinence	26 (59.1)	21 (67.7)	0.446^d^
Splinting or digitation for urination	10 (23.3)	4 (12.9)	0.262^d^
POP-Q grade (n) (%)			
2	23 (52.3)	14 (45.2)	0.544^d^
3	21 (47.7)	17 (54.8)	

BMI: Body mass index, CAD: Coronary Artery Disease, CHF: Congestive Heart Failure, COPD: Chronic Obstructive Pulmonary Disease, CRAIQ: Colorectal-Anal Impact Questionnaire, DM: Diabetes Mellitus, HT: Hypertension, PFIQ: Pelvic Floor Impact Questionnaire, POP-Q: Pelvic Organ Prolapse Quantification, POPIQ: Pelvic Organ Prolapse Impact Questionnaire, SD: Standard deviation, UIQ: Urinary Impact Questionnaire, *: statistically significant

^a^Independent sample t test

^b^Mann–Whitney U test

^c^Fisher’s exact test

^d^Chi-square test

**Table 2:** POP-Q measurements of the groups

	Group-1 (sitting) (n = 44)	Group-2 (squatting) (n = 31)	p
Aa (median) (min, max)	1 (− 1, 3)	1 (− 1, 3)	0.104
Ba (median) (min, max)	0 (− 2, 3)	1 (− 2, 4)	0.298
Ap (median) (min, max)	− 2 (− 1, − 3)	− 2 (− 1, − 3)	0.801
Bp (median) (min, max)	− 2 (− 1, − 3)	− 2 (− 1, − 3)	0.403
C (median) (min, max)	− 4 (− 1, − 7)	− 5 (− 2, − 6)	0.847
D (median) (min, max)	− 6 (− 3, − 7)	− 6 (− 4, − 7)	0.834
Gh (median) (min, max)	4 (2, 5)	4 (2, 4)	0.037
Pb (median) (min, max)	2 (1, 4)	2 (2, 3)	0.393
TVL (median) (min, max)	7 (5, 9)	7 (6, 8)	0.303

Data are shown as median (min, max). The Mann–Whitney U test was used for statistical analysis

Gh: genital hiatus, Pb: perineal body, TVL: total vaginal length

## Discussion

The critical factor in patients with POP deciding to pursue intervention measures is the worsening of the symptoms, rather than the anatomical progression of the prolapse [9]. In our study, the waiting time for Group 2 patients (squat position) to decide on surgery was shorter than that for Group 1 (sitting position) patients (p = 0.001). In addition, the PFIQ and POPIQ scores were higher in Group 2 patients than in Group 1 patients (p = 0.001, p < 0.001). In addition, when the pain scores of each group of patients were determined, the mean VAS score of the patients using the squatting position was significantly higher (p < 0.001). BMI, parity, age, smoking, and pad count were not significantly different between the groups; therefore, the increase in the symptom scores and VAS scores of the patients who used the squatting position may have affected their decision to undergo surgery earlier. In addition, no significant difference was found between the UIQ scores of the patients in either group. As a result of these values, it appears that toileting position does not affect the lower urinary tract symptom score in our patient groups. According to the Sakakibara study, defecation in the squatting position was observed to be more physiological than that in the sitting position [5]. In our study, no difference was observed in the Colorectal-Anal Impact Questionnaire scores among our patient groups (p = 0.797). More data may be needed to evaluate the effect of position on defecation symptoms in patients with POP and to make comparisons with the literature.

Various factors influence the toileting behavior of women, including social, cultural, and medical factors. In Western countries, the widespread use of sitting toilets began in the nineteenth century when sewage systems were developed to improve sanitation [10]. Women in various Asian and African countries void and defecate in a squatting position, whereas the sitting position is preferred in Western countries.

Pelvic floor health and IAP are essential components of voiding, defecation dysfunction, and POP etiopathogenesis in women. IAP changes and pelvic floor muscle load differ in the squatting and sitting positions. Studies have concluded that defecation is physiological in the squatting position; the pelvic floor muscles are more dilated [5]. Although some studies have argued that the squatting position is better for defecation, there is no consensus on whether the position affects voiding function. In addition, there is no study on the effect of toileting position on the natural course of POP.

AVWP, known clinically as cystocele, is the most common form of POP [11]. The anterior vaginal wall is also the area with the highest rate of primary and recurrent support defects [12]. Many risk factors for POP have been proposed, and the cause is most likely multifactorial. The magnitude of AVWP is sensitive to maximal abdominal pressure, and a decrease in the resistance of the levator ani muscle to stretching results in a larger hiatus size [13]. Squatting movements are also considered one of the most strenuous actions that result in increased abdominal pressure. Evidence has emerged that strenuous physical activity increases the risk of pelvic floor disorders, such as POP and urinary incontinence [14]. The definition of “strenuous” is primarily subjective: in the pelvic floor literature, strenuous usually refers to activities thought to significantly increase IAP [15]. There is no established, evidence-based maximum IAP threshold used to guide activity restriction for safety purposes. In laboratory studies, a safe threshold value of > 60 cmH2O is recommended as the maximum IAP. Activities that increase IAP above this threshold may be restricted [16].

Intrathoracic pressure (ITP), IAP, and the Valsalva maneuver (VM) play important roles in activities of daily life movements [17]. An IAP increase is needed to maintain balance during trunk movement. IAP is lowest when the trunk is in an isometric position. Increases in ITP and IAP initiated by the VM are considered body techniques that increase the stability of the body during physical activity [18]. IAP levels change in response to trunk asymmetry. During flexion–extension movement of the trunk, the pressure in the abdomen can increase to up to 150 mmHg. With body torsion, the IAP also increases [19]. Increasing IAP is achieved physiologically by reflexively contracting the anterior abdominal wall muscles to ensure trunk stabilization [20]. There is no study measuring IAP during toileting. However, as a result of the studies evaluating the effect of a squatting position on IAP, it can be expected that the IAP that occurs while performing a squat is higher than that when sitting [4, 17, 20].

Patients with POP generally also have a high BMI. An elevated BMI is an important factor in increasing IAP. Patients with high BMI may need to increase their IAP to maintain their balance while squatting. In addition, patients who perform voiding and defecation in a squatting position do so by opening their legs and squatting low enough to bring their knees to the shoulder level; this may cause the genital hiatus to open more than it does in the sitting position. In patients who defecate and micturate in a squatting position, increased IAP and greater opening of the genital hiatus may make it easier for the vaginal wall to exit the hymenal ring.

Evidence regarding the evolution of POP is scarce and conflicting [9]. Given that AVWP is a disease with minimal morbidity, it is crucial to understand the factors that increase the likelihood of patients choosing an intervention over observation. This will allow healthcare providers to provide more comprehensive counseling to women considering therapeutic measures for this disease. Vaginal swelling that the patient can see or feel is the most specific symptom of POP [9]. The complaint of disturbing vaginal swelling is associated with the final intervention decision. However, the intensity of symptoms rather than physical examination findings determines patient treatment preferences. Vaginal bulging was seen as the main symptom when deciding on surgery in most patients in our study. Specifically, vaginal bulging was the leading symptom in 96.8% of patients in Group 2, and it was the leading symptom in 84.1% of Group 1 patients. This difference may be related to the fact that the patient feels vaginal bulging more due to the increase in IAP while performing a squat and then standing up, rather than increasing their IAP after assuming a squatting position.

Most clinicians accept an association between AVWP and lower urinary tract dysfunction and often assume a close association between worsening AVWP and worsening urinary symptoms. The decision to intervene is consistent with changes or worsening of these associated conditions. In our study, it was observed that the rate of urgency and incontinence in Group 2 patients was higher than that in Group 1 patients. However, the rate of splinting or digitation for urination was lower in Group 2. The reason for this may be the balance problem that the patients may encounter in performing the digitation action in the squatting position.

In current practice, there are two active therapeutic interventions for POP: the use of an intravaginal pessary or surgical correction. A third option, which avoids surgical and anesthetic complications, is observation. When discussing these treatment options with a patient, a healthcare professional should consider the patient's preference, lifestyle factors, prolapse size, comorbidities, age, desires for future childbearing, and the risks and benefits of all treatment options. While informing the patient about treatment options, it is important to explain the lifestyle changes aimed at reducing the pressure on the pelvic organs. Among these recommendations, weight loss and avoidance of activities that increase IAP, especially in obese women, should come first. In addition, considering the data we obtained from our study, changing the toileting position may also be a lifestyle change option.

The present study has some limitations. IAP of the patients in squat and sitting positions were not measured with a rectal manometer. Furthermore, it was questioned if the patients were doing heavy housework such as lifting sofa or bed, but there is no data on the details of the what kind of housework.

## Conclusion

Voiding and defecation physiology are closely related to pelvic floor health. In patients with symptomatic POP, increased IAP while performing the squat position during defecation and voiding may increase the severity of patients' symptoms related to prolapse more than that of sitting position. Therefore, questioning the toileting position of patients with AVWP may help inform management decisions, with changing to a sitting position encouraged.

## Data Availability

The datasets used and/or analyzed in the current study are available from the corresponding author on reasonable request.

## Abbreviations

AVWP: Anterior vaginal wall prolapse
CRAIQ: Colorectal-Anal Impact Questionnaire
PFIQ: Pelvic Floor Impact Questionnaire
POPIQ: Pelvic Organ Prolapse Impact Questionnaire
UIQ: Urinary Impact Questionnaire
POP: Pelvic organ prolapse
IAP: Intra-abdominal pressure
ITP: Intrathoracic pressure
VM: Valsalva maneuver
